# Predictors of quality of life and resilience in patients with ovarian cancer during the COVID-19 pandemic: a cross-sectional study

**DOI:** 10.1007/s00404-024-07870-y

**Published:** 2024-12-17

**Authors:** Larissa Schilling, Anne Toussaint, Angelika Weigel, Dorothea Lewitz, Golo Aust, Jeanne Töllner, Gülten Oskay-Özcelik, Annette Hasenburg, Bernd Löwe, Barbara Schmalfeldt

**Affiliations:** 1 Department of Gynecology, University Medical Center Hamburg-Eppendorf, Hamburg, Germany; 2 Department of Psychosomatic Medicine and Psychotherapy, University Medical Centre Hamburg-Eppendorf, Hamburg, Germany; 3 Department of Gynecology, University Medical Center Charité, Berlin, Germany; 4 Department of Gynecology, University Medical Center Mainz, Mainz, Germany

**Keywords:** Quality of life, Resilience, Ovarian cancer, COVID-19

## Abstract

**Purpose:**

The aim of this cross-sectional study was to investigate the psychosocial burdens of patients with ovarian cancer during the COVID-19 pandemic.

**Methods:**

Ovarian cancer patients answered a quantitative survey assessing their resilience (BRS) and quality of life (FACT-G7) as well as clinical (first- vs. ≥ second-line treatment), demographic (age < 65 vs. ≥ 65 years) and COVID-19 pandemic-related psychosocial impairment, i.e. anxiety (GAD7); depression (PHQ2); global physical, mental, and social health (PROMIS items). Analyses of variance were applied to compare psychological impairment between patients on first- vs. ≥ second-line treatment and between patients aged < vs. ≥ 65 years at start of treatment. Multiple linear regression analyses were performed to evaluate predictors of patients' resilience and quality of life based on demographic, clinical, and psychosocial variables.

**Results:**

Most of the 93 patients rated their physical and mental health, and satisfaction with social activities as good. Eighty-seven (91.4%) were somewhat or very concerned about the pandemic. Patients on first-line therapy reported a better quality of life (*p* = 0.03) and better general health (*p* = 0.014) than those on at least second-line therapy. Patients < 65 years old reported significantly more concern about the pandemic than older patients (*p* = 0.008). Predictors of resilience were severity of anxiety (GAD-7) and mental health. Predictors of quality of life were general health, severity of depression (PHQ-2), and type of therapy.

**Conclusions:**

Patients in first line of treatment and younger patients could benefit from support in coping with pandemic-related burdens, meaning that attention should be paid to potential psychological distress, which should be treated alongside the cancer.

## What does this study add to the clinical work

**Table:** 

It provides a better understanding of the psychological distress experienced by ovarian cancer patients during the pandemic. We were able to identify patient-related factors associated with quality of life and resilience, and therefore to better identify vulnerable patient groups in order to strengthen their mental health and to pay attention to potential comorbid symptoms of depression and anxiety.	

## Introduction

The COVID-19 pandemic presented the world and healthcare systems with unforeseen challenges. The first cases in Germany were reported in January 2020 [1]. Patients who had already been ill were more clearly burdened by the pandemic, not only in terms of treatment situation but also in terms of psychological distress. This led to a significant reduction in the patients' overall well-being [2–4].

Jacome, Deshmukh, Thulasiraman et al. conducted a literature review of publications from around the world on the impacts of COVID-19 on ovarian cancer patients and found four main issues: delayed diagnosis, delayed treatment, alternative management (e.g., preference for oral therapies, virtual management), and reduced quality of life (higher rates of worry, depression, and anxiety) [5]. In addition, the COVID-19 pandemic was associated with increased symptoms of generalized anxiety (44.9%), depression (14.3%), psychological distress (65.2%), and COVID-19-related fear (59%) in a cross-sectional study of 15,704 German residents [2].

Ovarian cancer is usually diagnosed at an advanced stage in older people and is the gynecological cancer with the highest morbidity and mortality. First-line treatment usually consists of surgery, with the aim of removing all macroscopically visible tumor, followed by a platinum-based chemotherapy. Subsequent lines of treatment for recurrence usually consist of further chemotherapy. After these active forms of therapy, various maintenance therapies are available. Once these have been carried out, intensified follow-up care is usually provided. (cf. German therapy guideline) [6].

In our study, we examined the psychosocial impairment of patients with ovarian cancer and the factors affecting their quality of life and resilience during the COVID-19 pandemic. We also exploratively investigated whether patients experienced different levels of distress depending on their line of therapy and age of therapy and aimed to identify prognostic factors associated with quality of life and resilience. These factors may have general relevance for therapy outcomes in patients with ovarian cancer, not only in times of a pandemic.

## Methods

This cross-sectional observational study was conducted in collaboration with the health sciences team at the University of Chicago Department of Obstetrics and Gynecology, who had carried out a comparable study in their department [7]. All patients diagnosed with ovarian cancer at all stages (FIGO I–IV) in the clinical information system of the University Medical Center Hamburg- Eppendorf, Germany, who provided written informed consent were invited to answer to self-report questionnaires (15–20 min) either at their hospital appointments or by mail. Recruitment took place between June 2020 and April 2021 based on convenience and availability. Patients who did not speak German or for whom an adequate interpreter was not available were excluded. The following clinical data were collected by reviewing the patients’ medical records:

- FIGO stage [FIGO I—IV] and grading
- current therapy [active primary treatment (first treatment after diagnosis), active recurrence treatment (treatment after diagnosis of recurrence), maintenance therapy (all patients receiving maintenance therapy), follow-up care (all patients receiving intensified follow-up care)]
- line of therapy [first-line treatment (treatment before the occurrence of a recurrence) vs. ≥ second-line treatment (treatment after the occurrence of a recurrence)]
- age [patients < 65 years at start of treatment vs. patients ≥ 65 years at start of treatment]
- start of therapy [date]

### Instruments

- FACT-G7 (Functional Assessment of Cancer Therapy; 7 items) assesses health-related quality of life (HRQOL) using Likert scale from 0–4, in which some items must be reversed. Individual items are summed to produce a total score between 0 and 28. The reliability of the scale was 0.74 and validity for this patient group was proven in a general population sample [8].
- The GAD-7 (Generalized Anxiety Disorder Scale-7; 7 items) screens for generalized anxiety using a Likert-scale from 0 = “not at all” to 3 = “nearly every day”. Sum scores of 5, 10, and 15 points are taken as the cut-off points for mild, moderate, and severe anxiety, respectively. It has good reliability and is well validated [9, 10].
- The PHQ-2 (Patient Health Questionnaire-2) comprises two items from the PHQ-9 to screen for depression severity. Sum scores range from 0–6 points, with a cut-off score of 3 for a positive screening for depression [11]. The questionnaire is well-validated for different patient groups [12].
- The BRS (Brief Resilience Scale; 6 items) measures the patients resilience. Responses vary from 1–5, giving a range of 6–30 points. The sum score is divided by the total number of questions answered for each participant (1.0–2.99 = low resilience, 3.0–4.3 = normal resilience, 4.31–5.0 = high resilience) [13].
- Additionally, one question was adapted from the ELSA (English Longitudinal Study on Aging;) regarding loneliness since the COVID-19 pandemic vs. before. It is for example used to study changes in mental health and social relationships [14].
- New questions specific to COVID-19 were included regarding how behavior and concern changed during the pandemic and were drawn from the 2020 US National Women's Health Study (one item regarding concern, one item regarding behavior) [15]. Additionally we added one item from the Posttraumatic Stress Disorder Checklist (PTSD-Checklist) to assess feelings when reminded of the pandemic [16].
- Further items were added: living arrangements, marital status, personal contacts, general health, loneliness, changes in behavior, changes in sexual interaction, appointment and contact changes and whether patients felt upset when reminded of the pandemic. Additionally, there were questions about current treatment and two open-ended questions regarding changes in social interaction and experiences as patients during the COVID-19 pandemic.

Measures taken from PROMIS Global Physical and Mental Health Questionnaires are listed in Table 2. The study was conducted in accordance with the Declaration of Helsinki and with the approval of the local ethics committee (PV7348).

### Statistics

First, descriptive statistical analyses (mean and standard deviation, or median and range, or percentages) were used to assess the demographic, psychosocial, and clinical characteristics and effects of COVID-19 in our sample. Second, Mann–Whitney U Tests as a non-parametric statistical test due to the continuous data not being normally distributed were used to compare patients based on their line of therapy (first- vs. ≥ second-line therapy) and their age (< 65 vs. ≥ 65 years) in terms of general, mental, and social health as well as depression, anxiety, quality of life, resilience, and concern about the pandemic. Bonferroni correction was applied for multiple comparisons. Statistical significance was accepted at the p < 0.05 level. Effect size (Cohen’s d) were calculated in case of significant differences.

Last, multiple linear regression analyses were performed to evaluate predictors of patients' resilience and quality of life as dependent variables and demographic, clinical, and psychosocial variables as independent variables. Assumptions of linear multiple regression, including linearity, independence, homoscedasticity, and normality of the residuals, and of residuals were also assessed using diagnostic plots and statistical tests (Durbin-Watson test for independence, Breusch-Pagan test for homoscedasticity, Q-Q plot for normality). Collinearity among predictors was assessed using variance inflation factor (VIF), with a VIF value greater than 10 indicating potential multicollinearity issues. Variables with high multicollinearity were removed from the model. The MLR model was estimated using the ordinary least squares (OLS) method. The coefficients (β) were interpreted to understand the relationship between each independent variable and the dependent variable, holding other variables constant. Statistical significance of the predictors was evaluated using F-tests, with a p-value threshold set at 0.05 for significance. Regression analyses were conducted with complete cases only. Statistical analyses were conducted using IBM SPSS 27.

## Results

Initially, 97 patients agreed to participate in the study. Since two of them did not fulfil the inclusion criteria and two did not fill out the questionnaire correctly, a total of 93 patients participated in our study (see Table 1 for demographic and clinical characteristics). The mean age was 61.87 years (SD 12.42; range 34–90 years), while the mean age at diagnosis was 59.61 years (range 31–85). Sixty-one patients (65.6%) were diagnosed at stage III. The median time since initial treatment was 27.18 months (SD 32.25) at the time of assessment. Fifty-nine (63.4%) patients were undergoing first-line, 34 (36.6%) second-line therapy. Regarding the type of therapy, twenty-six (28.0%) patients were in active primary treatment, 20 (21.5%) in active recurrence treatment, 38 (40.9%) in maintenance therapy, and 9 (9.7%) in follow-up treatment. Regarding living situation, only 8 (8.6%) patients reported changes during the pandemic and 20 (21.5%) reported living alone. When asked about concerns, most patients (85, or 91.4%) indicated feeling somewhat or very concerned about the COVID-19 pandemic. Regarding loneliness, 65 (69.9%) patients had hardly ever felt lonely before the coronavirus outbreak. A total of 44 (47.3%) had felt somewhat or very lonely since the outbreak. Seventy-four (79.6%) patients reported no change in appointments and 79 (84.9%) patients reported no difficulties getting in touch with their primary care provider.

**Table 1:** Demographic Characteristics and Impacts of COVID-19 (n = 93)

Age	
Mean (SD)	61.87 (12.42)
Range	34–90
Age at diagnosis, years	
Mean (SD)	59.61 (12.82)
Range	31–85
Stage (FIGO, initial at time of first diagnosis)	
I	11 (11.8%)
II	3 (3.2%)
III	61 (65.6%)
IV	18 (19.4%)
Time since initial treatment, months^a^	
Mean (SD)	27.18 (32.25)
Range	1–180
Line of therapy^b^	
First line	59 (63.4%)
≥ Second line	34 (36.6%)
Ongoing therapy	
Active primary treatment	26 (28.0%)
Active recurrence treatment	20 (21.5%)
Maintenance therapy	38 (40.9%)
Follow-up care	9 (9.7%)
Who do you live with?	
Alone	20 (21.5%)
Spouse/partner	48 (51.6%)
Spouse/partner and child (ren)	16 (17.2%)
Child(ren)	2 (2.2%)
Retirement home	1 (1.1%)
Friend(s)	3 (3.2%)
Missing	3 (3.2%)
Has who you live with changed because of COVID-19?	
Yes	8 (8.6%)
No	78 (83.9%)
Missing	7 (7.5%)
How concerned are you about the coronavirus pandemic?	
Very concerned	39 (41.9%)
Somewhat concerned	46 (49.5%)
Not very concerned	8 (8.6%)
Not at all concerned	0 (0%)
Have your regularly scheduled appointments with your physician been canceled, rescheduled, or changed to telephone visits?	
Yes	10 (10.8%)
No	74 (79.6%)
Missing	9 (9.7%)
Have you experienced difficulty getting in touch with your primary care provider or your ovarian cancer team?	
Yes	6 (6.5%)
No	79 (84.9%)
Missing	8 (8.6%)
Before coronavirus outbreak, how often did you feel lonely?	
Hardly ever	65 (69.9)
Some of the time	23 (24.7)
Often	4 (4.3)
Missing	1 (1.1)
Since the coronavirus pandemic, do you feel lonely?	
Much more often than before	12 (12.9)
Somewhat more often than before	32 (34.4)
The same as before	45 (48.4)
Less than before	1 (1.1)
Much less than before	1 (1.1)
Missing	2 (2.2)

^a^Calculated from treatment start date to survey date

^b^Treatment phase at the time of the survey

Table 2 presents the descriptive results of the psychological assessment questionnaires. Most participants rated their general physical and mental health as good, most were satisfied with their social activities and relationships and reported normal levels of resilience (BRS sum score 3.33, SD 0.73) [13]. In the FACT-G7, we observed a mean value of 16.43 (SD = 3.68), with a range of 0–28, (the higher the score, the better the quality of life). With mean scores of 4.67 (SD = 4.15) in the GAD-7 and 1.23 points (SD 1.33) in the PHQ-2, patients reported mild levels of anxiety and did not screen positive for depression, respectively [9, 11].

**Table 2:** Measures Taken from PROMIS Global Physical and Mental Health Questionnaires

In general, how would you rate your physical health? (*n* = 89)	
Mean (SD)	3.40 (0.96)
Median (range)	3 (1–6)
In general, how would you rate your mental health, including your mood and your ability to think? (*n* = 91)	
Mean (SD)	2.95 (0.90)
Median (range)	3 (1–5)
In general, how would you rate your satisfaction with your social activities and relationships? (*n* = 90)	
Mean (SD)	3.04 (1.06)
Median (range)	3 (1–5)
FACT-G7 (*n* = 92)	
Mean (SD)	16.43 (3.68)
Median (range)	17 (6–23)
GAD-7 (*n* = 93)	
Mean (SD)	4.67 (4.15)
Median (range)	4 (0–17)
PHQ-2 (*n* = 92)	
Mean (SD)	1.23 (1.33)
Median (range)	1 (0–6)
BRS (*n* = 91)	
Mean (SD)	3.33 (0.73)
Median (range)	3.5 (1.50–5.00)

*BRS* Brief Resilience Scale, *FACT-G7* Functional Assessment of Cancer Therapy, *GAD-7* Generalized Anxiety Disorder-7, *PHQ-2* Patient Health Questionnaire-2.

Regarding subgroup differences, patients on ≥ second-line therapy reported significantly lower quality of life and significantly worse general health than patients on first-line therapy. Patients who were younger at the start of their treatment were more concerned about the pandemic than patients who were aged ≥ 65 years when starting treatment. The groups did not significantly differ in any of any other assessed parameters (see Table 3).

**Table 3:** Comparison between first- and ≥ second-line therapy as well as age at the beginning of therapy (< 65 vs. ≥ 65 years) for depression, anxiety, quality of life, resilience, and concern about the pandemic

Variable	1st-line therapy(*n* = 59)	≥ 2nd-line therapy(*n* = 34)	*p*-value	ES
Depression severity(PHQ-2), *M (SD)*	1.19 (1.42)	1.29 (1.17)	0.718	n.a
Anxiety severity(GAD-7), *M (SD)*	4.69 (4.22)	4.62 (4.09)	0.932	n.a
Quality of life(FACT-G7), *M (SD)*	17.07 (3.62)	15.35 (3.58)	**0.030**	− 0.48
Resilience(BRS), *M (SD)*	3.30 (0.74)	3.38 (0.73)	*0.6*00	n.a
Concern, *M (SD)*	1.68 (0.68)	1.65 (0.54)	0.822	n.a
General health, *M (SD)*	3.21 (0.93)	3.73 (0.94)	***0.0*14**	0.56
Mental health, *M (SD)*	2.98 (0.88)	2.88 (0.95)	0.610	n.a
Social health, *M (SD)*	2.91 (1.01)	3.26 (1.11)	0.125	n.a

Variable	Age at start of therapy < 65 years (*n* = 59)	Age at start of therapy ≥ 65 years (*n* = 34)	*p*-value	ES
Depression severity (PHQ-2), *M (SD)*	1.32 (1.40)	1.06 (1.20)	0.368	n.a
Anxiety severity (GAD-7), *M (SD)*	5.20 (4.12)	3.74 (4.09)	0.100	n.a
Quality of life (FACT-G7), *M (SD)*	16.41 (3.83)	16.48 (3.44)	0.923	n.a
Resilience (BRS), *M (SD)*	3.27 (0.71)	3.44 (0.78)	0.285	n.a
Concern, *M (SD)*	1.80 (0.61)	1.44 (0.61)	**0.008**	− 0.59
General health, *M (SD)*	3.46 (1.01)	3.30 (0.88)	0.448	n.a
Mental health, *M (SD)*	2.96 (0.98)	2.91 (0.75)	0.787	n.a
Social health, *M (SD)*	3.16 (1.20)	2.85 (0.74)	0.183	n.a

*M* mean, *SD* standard deviation, *ES* effect size (Cohen’s d), *PHQ-2* Patient Health Questionnaire, 2-item depression scale (range: 0–6), *GAD-7* generalized anxiety disorder scale, 7-item anxiety scale (range: 0–21), *FACT-G7* functional assessment of cancer therapy—General (range: 0–28), *BRS* brief resilience scale (range: 0–30), Concern about the pandemic (range: 1 (very) —4 (not at all), General Health “In general, how would you say your health is?” (range: 1 (excellent)—5 (poor)), Mental Health “In general, how would you rate your mental health, including your mood and your ability to think?” (range: 1 (excellent)—5 (poor)), Social Health “In general, how would you rate your satisfaction you’re your social activities and relationships?” (range: 1 (excellent)—5 (poor)), ES effect size (Cohen’s d), *n.a* not applicable, significant p-values printed in bold.

In further analyses, we compared our patients in terms of other potentially relevant variables such as stage FIGO I versus FIGO II–IV, active treatment vs. follow-up care, and living alone vs. not living alone. We did not, however, find any statistical differences between these groups in terms of general, mental, and social health, depression severity, anxiety severity, quality of life, resilience, or pandemic-related concerns.

We tested, via multiple regression analyses, which variables were significant predictors of health-related quality of life and resilience. For resilience, two predictors were statistically significant: anxiety severity and mental health status (global item), showing that the larger the anxiety severity (GAD-7) and the worse the mental health (global item), the lower the resilience (Table 4). The worse the general health and the higher the severity of depression while remaining under active treatment, the worse the quality of life (Table 4).

**Table 4:** Multiple linear regression analysis

Multiple linear regression analysis of predictors of resilience (BRS) (*n* = 87)^a^			
Predictor	*b (SE)*	*ß*	* p*
Depression severity (PHQ-2)	– 0.108 (0.68)	– 0.201	0.118
Anxiety severity (GAD-7)	– 0.052 (0.020)	– 0.303	**0.012**
General health (global item)	– 0.095 (0.083)	– 0.128	0.255
Mental health (global item)	– 0.208 (0.077)	– 0.266	**0.008**
Social health (global item)	– 0.007 (0.066)	– 0.010	0.919
Concern (1 item)	0.029 (0.102)	0.026	0.774
Health-related quality of life (FACT-G7)	– 0.007 (0.021)	– 0.038	0.726

Multiple linear regression analysis of quality of life (FACT-G7) (*n* = 86)^b^			
Predictor	*b (SE)*	*ß*	* p*
Depression severity (PHQ-2)	– 0.754 (0.340)	– 0.274	**0.030**
Anxiety severity (GAD-7)	– 0.001 (0.106)	– 0.001	0.995
General health (global item)	– 1.808 (0.368)	– 0.474	**0.000**
Mental health (global item)	0.283 (0.402)	0.071	0.483
Social health (global item)	– 0.206 (0.329)	– 0.060	0.534
Resilience (BRS)	0.064 (0.573)	0.021	0.911
Active therapy	1.952 (0.627)	0.268	**0.003**

*PHQ-2* = Patient Health Questionnaire-2 (range 0–6); *GAD-7* = General Anxiety Disorder Scale-7 (range 0–21); General Health = “In general, how would you say your health is?” (range: 1 (excellent)—5 (poor)); Mental Health = “In general, how would you rate your mental health, including your mood and your ability to think?” (range: 1 (excellent)—5 (poor)); Social Health = “In general, how would you rate your satisfaction you’re your social activities and relationships?” (range: 1 (excellent) -5 (poor))

^a^ Concern = “How concerned are you about the coronavirus pandemic?” (range: 1 (very concerned) – 4 (not at all concerned); FACT-G7 = Functional Assessment of Cancer Therapy-7 (range: 0–28); adjusted R^2^ = .407, df = (7, 79), F = 0.000; significant p-values printed in bold

^b^ BRS = Brief Resilience Scale (range 0–30); Therapy line = 1st-line therapy or 2nd-line and higher; Active Therapy = Active Therapy or Maintenance Therapy and Follow-up care adjusted R^2^ = .421, df = (8, 78), F = 0.000; significant p-values printed in bold

## Discussion

The present study aimed to examine psychosocial impairment, quality of life and resilience of patients with ovarian cancer during the COVID-19 pandemic, to compare levels of distress depending on patients’ line of therapy and age and to identify factors affecting quality of life and resilience.

Results overall indicated that most patients experienced their physical and mental health, and satisfaction with social activities as good, while nearly all were somewhat or very concerned about the coronavirus pandemic. The high levels of concerns were comparable to Frey, Ellis, Zeligs et al., who found that 88,6% of patients with ovarian cancer were very concerned about their cancer during the pandemic [17]. Likewise, quality of life (FACT-G7) was lower 16.43 (SD = 3.68) in our sample compared with the a pre-pandemic general US population-based sample and lower than in adult cancer patient samples [18], possibly as a result of the pandemic. Surprisingly, study participants expressed on average low to mild levels of anxiety and did not screen positive for depression. The GAD-7 mean score in this study was comparable to the pre-pandemic value found in a general female population in Germany [19]. In a German adult population during the COVID-19 pandemic, the estimated mean PHQ-2 scores ranged from 0.81 (95% CI: 0.73–0.90) to 1.29 (95% CI: 1.21–1.37) [20]. Further, nearly half of patients in our sample (47.3%) experienced a considerable increase in loneliness since the COVID-19 pandemic, a factor which was, amongst others, predictive of depression and anxiety symptoms in women with ovarian cancer in a previous study [21] and by April 2020, rates of depression and anxiety were double the pre-pandemic benchmarks (29%) and 17% of women had symptoms of traumatic stress [15]. One might hypothesize that patients in the present sample used learned coping strategies such as emotional support, self-care, hobbies, planning, positive reframing, religion, instrumental support or avoidance, which might have contributed to these low levels of depression and anxiety [22]. An alternative interpretation of our results might be that the present study sample was particularly resilient. However, mean BRS scores rather indicated a “normal” resilience level in the present sample [13], which was additionally slightly lower as compared to the sample of our American colleagues [7].

Impairments of patients diagnosed with ovarian cancer partially differed based on line of therapy and age. In detail, patients on ≥ second-line therapy rated their quality of life and general health significantly lower than those on first-line therapy. These results are supported by the PRIMA trial (2022), which analyzed 733 patients with ovarian cancer and might be explained by disease progression, which was found to have a negative impact on health-related quality of life in patients with ovarian cancer [23]. We further observed significant group differences in terms of concern about the Covid-19 pandemic, with patients < 65 years old at the time of diagnosis reporting significantly higher levels of concern than those ≥ 65 years old at the time of diagnosis (p = 0.008). A systematic review by Arden-Close, Gidron, Moss-Morris showed that anxiety and depression are more prevalent in younger ovarian cancer patients [24]. One might hypothesize that younger patient groups are less likely to expect to fall ill themselves, have had less confrontation with a life-threatening illness overall and are therefore more worried about it.

We found that factors associated with higher resilience were anxiety severity and self-rated mental health (i.e., the less anxious and the more stable in terms of mental health, the more resilient our patients perceived themselves to be). Briefly, knowing the factors that influence resilience and quality of life is essential for our ovarian cancer patients. Zhou, Ning, Wang et al. for example analyzed the quality of life of breast cancer survivors, and showed that resilience is an important mediator between coping styles, perceived social support and HRQoL [25]. In terms of quality of life, we found that depression severity, self-rated global health, and mode of treatment were associated factors (i.e., quality of life increased with lower depression severity, increasing general health, and not being in active treatment anymore). In order to evaluate the influence of the therapy type, no distinction was made between the different therapy lines with regard to maintenance therapy and follow-up care. Several interventions are known to improve mental health, cope with distress, build resilience and thereby improve quality of life, such as the CALM (Managing Chance and Living Meaningful) intervention [26, 27]. These interventions are certainly an important additional tool for improving quality of life and resilience and should be further evaluated in future studies.

Furthermore, most of the patients answering the questionnaire (79.6%) reported that regular appointments with their doctor had taken place (Table 1). Eckford, Gaisser, Arndt et al. also investigated changes in cancer treatment during the COVID-19 pandemic and found that most of the patients with cancer evaluated in their study did not experience a change in primary cancer treatment. Overall, only 12.9% reported a change (i.e. a postponement, cancellation, or other type of treatment/mode of communication, such as videoconferencing) to a scheduled treatment, examination, or care plan [28].

Results of the present study should be interpreted in light of the following limitations: First, the present cross-sectional observational study with 93 patients did neither comprise a comparison cohort nor longitudinal data from times before the COVID-19 pandemic. Second, the opportunity to answer to the study measures either within the study center or at home might have resulted in a selection bias and patients particularly concerned, worried or bothered by the Covid-19 pandemic might have been underrepresented. Nevertheless, using several validated questionnaires, this study managed to identify relevant psychological difficulties that patients with ovarian cancer are struggling with during the COVID-19 pandemic. It was possible to identify vulnerable patient groups and to show relevant predictors for better quality of life and resilience, which are not only highly relevant in times of a pandemic but also in general cancer care.

In the future, we need larger prospective studies to validate the results of our study and to investigate interventions to support ovarian cancer patients individually. This will allow us to adapt tools for everyday clinical practice, with a focus on vulnerable groups (e.g. younger patients, patients on ≥ second-line, or patients with less support from family and friends). In addition, future studies should also focus on digital support for patients, which will be important in a new pandemic, but also in general. Although guided interventions have so far been considered more effective in reducing stress, anxiety and fatigue, digital interventions can be supportive, which warrants further research into long-term effects [29].

## Conclusion

Despite the specific burden that the COVID-19 pandemic has entailed, ovarian cancer patients did not report reduced resilience and were generally satisfied with their quality of life. However, patients who were younger at diagnosis and those on second-line or higher treatment may require increased support and appear to be a vulnerable group. It should be a general goal of treatment to strengthen patients` mental health and pay attention to potential comorbid symptoms of depression and anxiety in order to ensure the highest possible resilience and quality of life for patients with ovarian cancer.

## Data Availability

In accordance with the journal’s guidelines, we will provide our data for independent analysis by a team selected by the editorial team for the purpose of additional data analysis or for the reproducibility of this study at other centers if such is requested.

## Abbreviations

BRS: Brief Resilience Scale
COVID-19: Coronavirus SARS-CoV-2
ELSA: English longitudinal study on aging
FACT-G7: Functional assessment of cancer therapy
FIGO: Fédération Internationale de Gynécologie et d'Obstétrique
GAD7: Generalized anxiety disorder scale-7
GMH: Global mental health
GP: General practitioner
GPH: Global physical health
HRQOL: Health-related quality of life
PHQ-2: Patient health questionnarie-2
PROMIS: Patient-reported outcomes measurement information system
PTSD- Checklist: Posttraumatic stress disorder checklist
VIF: Variance inflation factor
OLS: Ordinary least squares
UKE: Universitiy Medical Center Hamburg–Eppendorf
